# Peptidomics and Computational Docking Prioritize Candidate Peptide–Protein Associations Relevant to Upper-Gastrointestinal Biology in a Fermented Soy-Derived Postbiotic

**DOI:** 10.3390/metabo16100794

**Published:** 2026-10-19

**Authors:** Annie Tremblay, Chase Harms, Amit Nankar, Ravi Gupta, Alex Buko, Thomas A. Tompkins

**Affiliations:** 1 Lallemand Bio-Ingredients, 1620 Préfontaine, Montreal, QC H1W 2N9, Canada; ttompkins@lallemand.com; 2 Zero State Inc., Phoenix, AZ 85050, USA; chase@zero-state.co (C.H.); amit@zero-state.co (A.N.); ravi@zero-state.co (R.G.); 3 Human Metabolome Technologies, Boston, MA 02134, USA; alex.buko@humanmetabolome.com

**Keywords:** soy peptides, postbiotic, peptidomics, gastroprotection, computational docking, Gastro-AD

## Abstract

**Highlights:**

**What are the main findings?**

- GAD contains multiple families of overlapping soy-derived peptides characterized by extensive truncation ladders.
- A diversity-sampling peptide panel was linked computationally to candidate interactors spanning mucosal immune regulation, extracellular-matrix remodeling, epithelial turnover, and tissue restitution.

**What are the implications of the main findings?**

- The findings support a multi-component model in which several peptide families may contribute to the biological properties of GAD, with mucosal integrity, injury responses, and tissue restitution emerging as priorities for future investigation.
- The integrated peptidomics and computational workflow provides a systems-oriented framework for investigating complex fermented peptide mixtures.

**Abstract:**

**Background/Objectives**: Gastro-AD^®^ (GAD) is a fermented soy-derived postbiotic supported by clinical and preclinical evidence related to gastric comfort and mucosal injury. Although fermentation-derived metabolites have been characterized, the peptide fraction remains poorly defined. This study characterized GAD peptides and explored their potential biological contexts. **Methods**: Untargeted peptidomics was used to identify soy-derived peptides in GAD and map them to source proteins. A diversity-sampling panel of ten peptides was selected using sequence, physicochemical, and predicted structural features. These peptides were screened against a human protein-structure library by computational docking, followed by GO enrichment and context-based prioritization of candidate interactors. **Results:** A total of 219 peptides, originating predominantly from soybean storage proteins, were identified. The GAD peptide fraction contained extensive overlapping sequence families and truncation ladders, consistent with extensive proteolytic processing. Three literature-reported antioxidant peptide sequences were identified as exact contiguous regions within longer GAD peptides. Large-scale docking against the screened human protein-structure library combined with GO-enrichment recurrence analysis and an independent review of top-ranked docking-derived candidates identified 180 candidate protein targets spanning innate immune and inflammatory defense, protease activity and peptide processing, extracellular-matrix remodeling, tissue repair and wound healing, and apoptotic signaling and epithelial turnover. Repeated randomized-affinity controls yielded substantially fewer FDR-significant GO terms than the observed docking-derived rankings, supporting non-random functional structure in the docking-derived protein rankings without establishing the accuracy of individual peptide–protein interactions. Context-based prioritization identified 43 unique Tier 1 and Tier 2 candidates with direct or context-dependent extracellular accessibility. The prioritized biological contexts included mucosal immune regulation, extracellular-matrix remodeling, epithelial turnover, and tissue restitution. **Conclusions**: The GAD peptide fraction described herein comprises multiple overlapping peptide families whose prioritized biological contexts center on mucosal defense, remodeling, and tissue restitution. These findings support further investigation of peptides as potential contributors to the multi-component biological properties of GAD.

## 1. Introduction

Plant-derived bioactives have long been investigated for their capacity to support gastrointestinal health. However, the traditional approach aiming to link biological effects to a single active compound has clear limitations when applied to complex postbiotic mixtures [1,2]; such reductionist approaches may overlook contributions from multiple molecular constituents. Growing evidence supports a multi-component model in which several compounds, including those present at relatively low concentrations, act through shared or complementary pathways and collectively contribute to the biological properties of plant-based preparations [2,3]. This perspective is particularly relevant for gastric comfort, a multifactorial physiological outcome involving interconnected processes such as epithelial integrity, immune regulation, sensory and neuromuscular function, and redox homeostasis [4,5,6,7].

Gastro-AD^®^ (GAD) is a plant-derived postbiotic produced through the controlled fermentation of soy flour (*Glycine max*) with the proprietary strain *Lactobacillus delbrueckii* R0187. The preparation was originally evaluated as Gastropharm before its subsequent development as GAD. Earlier Gastropharm studies included individuals with gastritis and gastric or duodenal ulcers and reported improvements in pain and associated dyspeptic symptoms [8,9,10], whereas more recent GAD studies have primarily evaluated gastric comfort and symptoms that overlap with those experienced in functional dyspepsia, including epigastric or abdominal discomfort, bloating, belching, fullness, nausea, and heartburn [11,12,13,14]. These clinical observations are complemented by preclinical studies in which the complete fermented-soy preparation reduced ethanol/HCl-induced gastric injury, inflammatory and apoptotic signaling, and acid-secretory responses in rats, and decreased squamous gastric-ulcer scores in horses [15,16]. Because these studies evaluated the complete formulation, their findings cannot be attributed to a single constituent or molecular fraction. Rather, the diverse clinical and preclinical observations support investigation of GAD as a multi-component preparation whose overall activity may reflect contributions from several peptide families as well as fermentation-derived metabolites.

Published NMR profiling, complemented by targeted HPLC and GC analyses, demonstrated that the controlled fermentation of soy flour substantially influenced the small-molecule composition of GAD, including the conversion of glycosylated soy isoflavones to their aglycone forms and changes in organic acids, amino acids, betaine, sterols, and related metabolites [17]. More comprehensive metabolomic characterization remains warranted to define this molecular fraction further. The present study addresses a complementary knowledge gap by characterizing the soy-derived peptide fraction. These analytical approaches examine distinct components of the same formulation and should not be interpreted as assigning product-level outcomes exclusively to either metabolites or peptides.

Peptides derived from plant proteins are increasingly recognized as important contributors to gastrointestinal physiology [18]. Beyond their nutritional role, food-derived peptides can exert local effects in the digestive tract, including modulation of oxidative stress, regulation of inflammatory signaling and mucosal barrier integrity [19,20,21,22,23]. Notably, these activities often do not rely on high systemic bioavailability, but rather on local, luminal or mucosa-proximal actions, making them especially relevant to clinical endpoints such as gastric and intestinal comfort [19]. As with plant metabolites, food-derived peptide bioactivity may be distributed across multiple sequences rather than attributable to a single peptide. Structurally related peptides can share sequence motifs and physicochemical characteristics relevant to biological activity, providing a rationale for grouping peptides into families and prioritizing representative sequences for further investigation [19,22,24,25].

The present study was designed to initiate characterization of the GAD peptide fraction using an exploratory workflow combining untargeted peptidomics and computational docking. We sought to characterize the identified soy-derived peptide profile by prioritizing candidate protein interactors and broader upper-GI biological contexts for future investigation.

## 2. Materials and Methods

### 2.1. Sample Preparation (Peptide Scan Advanced)

Gastro-AD^®^ (GAD; Lallemand Bio-Ingredients, Montreal, QC, Canada) samples were sent to Human Metabolome Technologies (HMT, Boston, MA, USA). Two aliquots from each of two independent soy-flour manufacturing lots (Lots 220328932 and 220419021) and two aliquots from each of four independent GAD manufacturing lots (Lots 66702278, 66702333, 66702335, and 66703328) were submitted for peptidomics analysis. Equal amounts of the submitted soy-flour samples were combined to generate a soy-flour pool, and equal amounts of the submitted Gastro-AD samples were combined to generate a GAD pool. Equal aliquots of the two group-specific pools were subsequently combined to generate a total pooled sample. The soy-flour, GAD, and total pools were subjected to MS/MS-based peptide identification.

Peptidome extraction, measurement, and data analysis were performed using HMT’s Peptide Scan Advanced service. A total of 100 μL of 0.1% formic acid was added to 10 mg of each sample. After mixing and centrifugation at 2000× *g* for 1 min, the supernatant was used as the sample extract. Each 50 µL sample extract was then mixed with 10 µL of internal standard solution and 300 µL of 85% acetonitrile (*v*/*v*) containing 0.1% formic acid (*v*/*v*), followed by centrifugation at 14,000× *g* for 5 min at 20 °C. After centrifugation, 250 µL of the supernatant was transferred to a low-protein-binding tube. The supernatant was dried, and the residue was reconstituted in 100 µL of 20% acetonitrile (*v*/*v*) containing 0.1% formic acid (*v*/*v*), then applied to a solid-phase extraction column (MonoSpin C18, 5010-2170, GL Sciences Inc., Torrance, CA, USA). After desalting with 0.1% formic acid in water, peptides were eluted with 100 µL of 60% ethanol (*v*/*v*) containing 0.1% formic acid (*v*/*v*). The eluate was then dried, and the residue was reconstituted in measurement solutions containing 25 mM NDSB-201, 25 mM NDSB-256, and 1 mM SB-14 for analysis.

### 2.2. Peptidome Analysis (Peptide Scan Advanced)

Peptidome analysis was conducted according to HMT’s Peptide Scan Advanced package, using capillary electrophoresis–Fourier transform mass spectrometry (CE-FTMS). Briefly, CE-FTMS analysis was carried out using an Agilent 7100 CE capillary electrophoresis system coupled to an Orbitrap Exploris 240 mass spectrometer (Thermo Fisher Scientific Inc., Waltham, MA, USA), an Agilent 1260 isocratic HPLC pump, an Agilent G1603A CE-MS adapter kit, and an Agilent G1607A CE-ESI-MS sprayer kit (Agilent Technologies, Inc., Santa Clara, CA, USA). The systems were controlled by Agilent MassHunter Workstation Data Acquisition software (Agilent Technologies) and Xcalibur software (Thermo Fisher Scientific) and connected by a fused silica capillary (50 μm i.d. × 80 cm total length) with a background electrolyte consisting of 40% MeOH (*v*/*v*) and 1 M formic acid. The spectrometer was scanned from *m*/*z* 300 to 4500, and peptide identification was performed using data-dependent acquisition (DDA) mode. Specifically, an MS1 scan range of *m*/*z* 300–4500 was used at a resolution of 60,000, after which the top 20 ions in each spectrum were selected for MS/MS analysis at a resolution of 15,000. MS/MS spectra were analyzed using PEAKS Studio 13 (Bioinformatics Solutions, Waterloo, ON, Canada) to identify peptides. MS/MS spectra were searched against UniProt Release 2025_04 using proteome ID UP000008827, comprising 74,859 searched entries. Precursor and fragment mass-error tolerances were set to 10 ppm and 0.02 Da, respectively. No enzyme specificity was imposed, and the search was performed in unspecific-digestion mode for peptides ranging from 6 to 45 amino acids. Deep Learning Boost was not applied. Peptide-spectrum matches (PSMs) were filtered at a 5% false discovery rate (FDR), with no additional peptide-level filter. Protein groups were filtered at a 1% FDR and required at least one unique peptide. The FDR for peptide-spectrum matches was set to 5% because the unspecific-cleavage search required for endogenous peptidomics substantially expands the search space and because applying a conventional 1% proteomics threshold under these conditions may excessively reduce the recovery of potentially valid endogenous peptide identifications.

Only pooled samples were subjected to MS/MS-based identification, and the peptides identified from these pooled samples were subsequently annotated in the MS1 data of individual lots using tolerances of ±5 ppm for *m/z* and ±0.2 min for migration time. Peaks were extracted using MasterHands, automatic integration software (Keio University, Tsuruoka, Yamagata, Japan). Areas of the annotated peaks were then normalized to the internal standards and sample volume in order to obtain the relative level of each peptide. Peptide relative signals were summarized descriptively across the independent manufacturing lots using group means, GAD-to-soy-flour ratios, and detection status. PCA of the 219 identified peptides was used to visualize overall lot-level peptide-profile variation. No peptide-level inferential statistical testing was performed. Annotation in an individual lot indicates detection of a corresponding MS1 feature within these criteria and does not constitute independent MS/MS sequence confirmation in each manufacturing-lot replicate. The complete peptide-level dataset, including lot-level relative signals and detection status, is provided in Table S1.

### 2.3. Principal Component Analysis 

Principal component analysis was performed using the relative signal profiles of the 219 identified peptides. The two soy-flour and four GAD manufacturing lots were treated as observations, and the identified peptides were treated as variables. Scores for the first two principal components were plotted to visualize overall variation among the lot-level peptide profiles.

### 2.4. Representative Peptide Selection

The library of soybean-derived GAD peptides was assembled for computational prioritization. The final analysis set consisted of 219 peptide sequences after duplicate removal, amino-acid sequence validation, and assignment of unique peptide identifiers.

To select a tractable set of representative peptides while preserving chemical and structural diversity across the library, peptides were clustered using a combined sequence, structure, and physicochemical feature representation. Peptide structures were predicted using OpenFold3-preview2 [26] with the openfold3-p2-155k checkpoint, template search enabled, MSA search enabled, and a fixed random seed of 42. Sequence-derived features included peptide length, amino-acid composition, reduced amino-acid group composition, and reduced dipeptide-frequency descriptors. Physicochemical descriptors included molecular weight, aromaticity, instability index, predicted isoelectric point, GRAVY hydrophobicity, secondary-structure propensity estimates, and a net charge-like descriptor. Structure-derived descriptors were calculated from predicted C-alpha coordinates and included radius of gyration, end-to-end distance, principal-axis ratios, pairwise C-alpha distance distributions, local bend-angle distributions, nonlocal contact fractions at multiple distance thresholds, and mean and standard deviation of the structure confidence/B-factor field.

Because the analyzed peptides are short sequences (6–45 amino acids), the predicted structures were treated as computational conformational representations rather than as experimentally established solution structures. Short peptides may be conformationally flexible, intrinsically disordered, or adopt different conformations upon interaction with a binding partner; consequently, a single predicted free-peptide structure may not represent the complete biologically accessible or receptor-bound conformational ensemble. Structural descriptors derived from the OpenFold3 predictions were therefore used as one component of the peptide feature representation rather than as definitive structural assignments. To assess whether representative-peptide selection depended strongly on these predicted structural features, clustering sensitivity analyses were additionally performed using sequence and physicochemical descriptors while excluding the predicted structural descriptors. Alternative peptide conformations were not propagated through the proteome-scale docking analysis, and this limitation was considered when interpreting individual predicted peptide–protein interactions.

Numeric features were median-imputed where necessary, near-constant features were removed, and the remaining features were standardized. Principal component analysis was used to retain 95% of feature variance. UMAP was then used, when available, to generate a lower-dimensional embedding for clustering and visualization. Peptides were clustered using Gaussian mixture models, with the number of clusters and covariance model selected by Bayesian information criterion across candidate models. This procedure separated the 219 peptides into 10 clusters. For each cluster, the medoid peptide, defined as the peptide with the smallest mean distance to other members of the cluster in the clustering embedding, was selected as the representative peptide. These 10 representative peptides were used for downstream proteome-scale docking.

### 2.5. Clustering and Representative-Panel Sensitivity Analyses 

Because the reduction of 219 peptides to 10 representatives constitutes a substantial dimensionality-reduction step, post hoc sensitivity analyses were performed to evaluate the robustness of the clustering solution and the coverage provided by the selected representative panel. Clustering was repeated under alternative features and embedding conditions, including PCA-only clustering without UMAP and clustering using sequence and physicochemical descriptors without predicted structural features. Sensitivity to UMAP stochasticity was assessed across independent random seeds, and cluster stability was further evaluated by repeated resampling of the peptide dataset. Agreement between alternative cluster assignments was quantified using the adjusted Rand index (ARI). Because exact cluster assignments and medoid identities were not expected to be invariant across alternative representations, representative-panel performance was also evaluated independently of cluster labels. The 10 selected peptides were compared with 10,000 randomly sampled 10-peptide panels by measuring the distance from every peptide in the 219-peptide library to its nearest selected representative in standardized feature space. This analysis was performed in both the complete sequence–structure–physicochemical representation and a structure-independent sequence–physicochemical representation. The representative peptides were therefore interpreted as a diversity-sampling panel rather than as definitive biological proxies for every member of their assigned cluster.

### 2.6. Human Protein Structure Library Construction

A human protein structure library was generated from experimentally determined structures in the RCSB Protein Data Bank accessed in April 2026 [27]. Candidate polymer entities were retrieved using an RCSB Search API query requiring Homo sapiens source organism, protein polymer type, RCSB 70% sequence-identity cluster membership, experimental determination by X-ray diffraction or electron microscopy, combined experimental resolution of 2.5 A or better, and polymer sequence length of at least 50 residues. The query returned all matching polymer-entity hits. Candidate structures were then prioritized using selected-chain length and experimental resolution, producing an approximately 5000-protein human structure library for peptide docking.

For each retained structure, all selected protein chains were extracted for docking. Bound ligands, crystallographic waters, ions, and cofactors were removed, and receptor structures were protonated at pH 7 prior to docking. This uniform preparation procedure was used to provide a consistent receptor representation across the structurally heterogeneous protein library. The resulting structures should therefore be considered standardized ligand- and cofactor-depleted receptor representations rather than complete reconstructions of the native holo state of every target. For proteins whose binding-site geometry or electrostatics depend strongly on metal ions, cofactors, prosthetic groups, or tightly bound waters, this preparation may reduce the physiological realism of the predicted interaction. PDB identifiers, polymer-entity identifiers, chain identifiers, structure metadata, and downstream gene annotations were retained to allow docking results to be traced from structure-level predictions to gene-level enrichment analysis.

### 2.7. Peptide–Protein Docking Screen

Peptide-protein docking was performed using DiffPepDock v1 [28] with the diffpepdock_v1.pth model weights [29]. The 10 representative peptides were docked against the approximately 5000-protein human structure library in chunked receptor batches suitable for parallel execution on GPU nodes. For each peptide-receptor pair, four docked pose samples were generated using 150 diffusion timesteps, a minimum diffusion time of 0.01, center-position noise enabled, center-position noise standard deviation of 1.0, and noise scale of 0.5.

Docked poses were scored using the PRODIGY [30] command-line implementation incorporated into the docking post-processing workflow. PRODIGY was used to generate a structure-based predicted binding free-energy estimate for each docked complex. For the primary structure-library screen, the most favorable of the four pose-level PRODIGY estimates was retained as the receptor-level ranking score for each peptide–receptor pair, with more negative values ranked as more favorable. These values were used as relative screening and prioritization scores and were not interpreted as experimentally determined binding affinities.

Because selecting the most favorable value from a small pose ensemble can introduce optimistic selection bias, a post hoc pose-aggregation sensitivity analysis was performed using the previously generated pose-level results for Peptide 4. Receptor rankings were independently reconstructed using the median and arithmetic mean of the four PRODIGY estimates. Agreement with the primary best-of-four ranking was assessed using Spearman rank correlation, Kendall rank correlation, and overlap among the highest-ranking receptor sets. The downstream GO Biological Process enrichment and ranking-depth convergence workflow was also repeated using the median- and mean-based receptor rankings. No additional docking was performed for this sensitivity analysis.

### 2.8. Mapping PDB Identifiers to Gene Identifiers

Ranked docking hits were mapped from PDB identifiers and chain identifiers to corresponding human gene identifiers using UniProt [31] accessions obtained through the PDBe SIFTS API [32]. Chain-specific annotations were used when multiple chains or genes were associated with a PDB entry. Only entries mapping to human UniProt accessions were retained for gene-level enrichment analysis. Gene identifiers were deduplicated where required for enrichment testing, while PDB-, chain-, and protein-level records were retained for target traceability. The resulting ranked gene lists were used as input for Gene Ontology enrichment analysis.

### 2.9. Gene Ontology Enrichment Analysis

Gene Ontology enrichment analysis was performed using PANTHER [33] to identify biological processes overrepresented among the highest-ranked predicted peptide-interaction candidates. For each representative peptide, docking-derived protein targets were converted to gene identifiers and analyzed using the PANTHER GO Biological Process Complete annotation set (Gene Ontology term GO:0008150) for *Homo sapiens*, following the approach described by Harms and Klein-Seetharaman [34]. Overrepresentation was tested using Fisher’s exact test with false-discovery-rate (FDR) correction for multiple testing. Terms were retained as significant when both the nominal *p* value and FDR-adjusted *p* value were <0.05. The reference set for all enrichment analyses consisted of the unique human proteins represented in the complete screened docking library, rather than the entire human proteome, so that enrichment reflected preferential representation within the structural protein search space used for docking. Ranking depth was defined at the PDB/chain-row level, after which gene identifiers were deduplicated before submission to PANTHER.

To define a reproducible ranking depth for downstream enrichment interpretation, GO enrichment was evaluated from *N* = 25 to *N* = 505 top-ranked PDB/chain entries in increments of 10. At each ranking depth, significant GO Biological Process terms were ranked by fold enrichment, and the top 25 terms, or all significant terms when fewer than 25 were available, were retained. Jaccard similarity was calculated between the GO-term sets obtained at successive ranking depths for which at least one significant term was present. Local convergence was defined operationally as a Jaccard similarity of at least 0.80 across three consecutive valid transitions, and the earliest ranking depth satisfying this criterion was selected for downstream analysis. The 0.80 threshold was chosen to require high overlap between successive GO-term sets, while the three-transition requirement was used to reduce selection based on a single transient similarity between adjacent ranking depths. These parameters were used as an operational ranking-depth heuristic and were not calibrated against biological ground truth or interpreted as evidence that the underlying docking predictions were correct. Sensitivity to the convergence definition was evaluated by varying the Jaccard threshold and the number of consecutive transitions required. Complete ranking-depth trajectories were retained for assessment of convergence behavior.

An absolute PRODIGY score cutoff was not imposed because the predicted energy estimates were used for relative ranking across a structurally heterogeneous receptor library and no experimentally calibrated universal peptide–protein score threshold was available for this analysis. Selecting such a threshold after inspection of GO results would itself introduce an outcome-dependent cutoff. The rank-based convergence procedure was therefore used only to define a reproducible subset for downstream comparison. Additional methodological details, complete ranking-depth trajectories, and convergence-definition sensitivity analyses are provided in the Supplementary Methods, Figure S1, and Table S3A–D.

### 2.10. Negative Control: Permutation Test for GO Enrichment

To assess whether significant GO enrichment could arise from arbitrary associations between docking-derived scores and protein identities, a repeated randomized-affinity negative-control analysis was performed independently for each representative peptide. For each peptide, the ranking depth N previously selected from the observed GO-profile convergence analysis was held fixed. The affinity scores across the complete screened receptor set were randomly reassigned among protein identities, thereby preserving the empirical score distribution while disrupting the association between predicted score and receptor identity. Each randomized receptor list was reranked from most to least favorable predicted score, and the top N entries were subjected to the same PANTHER GO Biological Process overrepresentation analysis used for the observed dataset.

For every randomized dataset, enrichment was performed using Homo sapiens, GO Biological Process Complete (GO:0008150), Fisher’s exact overrepresentation test, FDR correction, and the complete screened docking library as the custom reference background. GO terms were considered significant when both the nominal *p* value and FDR-adjusted *p* value were <0.05. Five hundred independent randomized rankings were evaluated for each representative peptide.

The primary null statistic was the number of FDR-significant GO Biological Process terms recovered at the observed ranking depth. The empirical permutation *p* value was calculated as:

$$P_{\mathrm{emp}}\;=\;\frac{1\;+\;n_{\mathrm{null}\geq \mathrm{obs}}}{B\;+\;1}$$

where *B* = 500 and *n_null ≥ obs_* is the number of randomized rankings yielding at least as many significant GO terms as the corresponding observed peptide profile. This analysis was performed separately from the Jaccard-based ranking-depth convergence procedure: the convergence criterion was used only to select the ranking depth, whereas the permutation analysis tested whether enrichment of comparable magnitude could arise after disruption of the docking-score-to-protein association.

### 2.11. Comparison of Enrichment Profiles Across Representative Peptides

To evaluate whether docking-derived biological profiles were consistent across representative peptides, GO-term enrichment profiles were compared across all 10 representative peptides.

For each GO term, the number of representative peptides whose convergence-selected enrichment profile included that term was counted. GO terms were ranked by the number of peptides in which they appeared (recurrence), with ties broken by a composite recurrence score incorporating the fraction of peptides, mean fold enrichment, and median significance across peptides. Pairwise Jaccard similarities were also calculated between enriched GO-term sets and between convergence-selected top-N PDB/protein sets. GO terms recurring across more than one peptide representative were then used as a prioritization framework to extract candidate interactors for further biological annotation.

### 2.12. Identification of Relevant Peptide Groups and Protein Targets

Candidate proteins were prioritized through two complementary and independently traceable routes. In the first, GO-guided route, GO Biological Process terms were considered recurrent when they occurred in the convergence-selected enrichment profiles of at least two representative peptides. Recurrent terms were reviewed for relevance to the biological domains considered in this study, including epithelial repair and mucosal integrity, inflammation and immune signaling, oxidative stress regulation, extracellular-matrix remodeling, protease signaling and protein turnover, nutrient and amino-acid metabolism, and tissue remodeling/wound healing. For each retained GO term, the PANTHER geneinfo API was used to retrieve protein-level annotations for proteins within each representative peptide’s convergence-selected top-N receptor set. A protein was retained through the GO-guided route only when it was present within the relevant convergence-selected receptor set, and its PANTHER-returned annotations explicitly included the corresponding recurrent GO term. This produced a direct and auditable mapping from representative peptide to ranked receptor, GO term, and candidate protein.

In the second, top-ranked docking route, the 10 PDB/chain entries with the most favorable PRODIGY-predicted binding free-energy estimates for each representative peptide were examined independently of GO-term membership. These candidates were reviewed for documented or biologically plausible relationships to the same functional domains used in the GO-guided analysis. This route was included to retain highly ranked peptide-specific candidates that could be missed by a recurrence-based GO filter.

Candidate proteins identified through either route were consolidated into a nonredundant set while retaining their identification route, associated representative peptide, PDB/chain entry, and GO-term information, where applicable. The complete candidate set is provided in Table S2. Biological review was used to contextualize and classify the computationally prioritized candidates and should not be interpreted as experimental validation of the predicted peptide–protein interactions.

### 2.13. Classification of Candidate Interactors into Biological Domains

Candidate interactors were classified using a rule-based procedure incorporating annotated protein function and GO associations, upper-GI biological relevance, tissue and subcellular localization, and plausible peptide accessibility. Targets were assigned to Tier 1 when directly accessible in the gastric lumen; Tier 2 when extracellular accessibility depended on secretion, degranulation, proteolytic activation, receptor topology, plasma extravasation, or mucosal injury; and Tier 3 when accessibility required peptide internalization or when upper-GI relevance, localization, or structural context remained indirect or uncertain. For nonredundant visualization, each Tier 1 or Tier 2 target was assigned to one primary functional domain according to its predominant annotated biological function. All assignments were reviewed and finalized by the authors.

## 3. Results

### 3.1. Peptidome Profile of Gastro-AD

PCA of the 219 identified peptide features (Figure 1) showed visual separation between the soy-flour and Gastro-AD^®^ (GAD) peptide profiles along the first principal component (F1), which accounted for 75.96% of the total variance. The second principal component (F2) accounted for an additional 17.36%, such that the first two components together explained 93.32% of the observed variance. The two soy-flour manufacturing lots showed closely aligned profiles, whereas greater dispersion was observed among the four GAD manufacturing lots. The PCA was interpreted descriptively.

Fermentation with *L. delbrueckii* R0187 produced a peptide relative-signal profile that differed descriptively from that of unfermented soy flour (Figure 2).

Across the 219 identified sequences, peptides ranged from short oligopeptides to longer fragments exceeding 30 amino acids. Peptide mapping identified 59 distinct UniProt accessions as potential source proteins. These accessions clustered into a limited number of biologically related to six major protein families, with soybean storage globulins, particularly glycinin and β-conglycinin, accounting for much of the mapped peptide diversity. Additional peptides were assigned to lectins, protease inhibitors, 7S basic globulins, oleosins, and other soybean proteins; the complete peptide-level results are provided in Table S1. Untargeted MS/MS analysis revealed that GAD was characterized by a large number of overlapping peptide sequences, differing by limited N- or C-terminal truncations. Rather than isolated, unique peptides, the dataset was dominated by peptide ladders, consistent with extensive proteolytic processing, although the specific enzymes and cleavage pathways involved were not determined (Figure 3).

### 3.2. Mapping Peptides to Potential Functions Through Literature Searches

#### Antioxidant and Redox-Associated Peptide Family

To examine whether the dataset contained peptides previously associated with antioxidant or redox-modulating activity, the literature was surveyed with a focus on soy-derived peptides for which experimental antioxidant activity has already been reported. Three peptide sequences previously reported to display antioxidant activity were identified as exact contiguous regions within longer GAD peptides: VNPESQQGSPR, LVPPQESQ, and EITPEKNPQ [35,36,37,38]. Each sequence showed 100% identity across the complete literature-reported peptide, although the corresponding GAD peptides contained additional N- and/or C-terminal residues. EITPEKNPQ occurred within peptides mapped to three soybean source proteins. Because the experimentally evaluated peptides were the shorter free sequences, the antioxidant activity of the longer GAD peptides cannot be inferred directly from sequence inclusion.

### 3.3. Mapping Peptides to Potential Functions Using Computational Docking

To reduce redundancy and capture shared biochemical features within the peptidome, peptides were grouped into families based on physicochemical similarity (Figure 4). This strategy is consistent with emerging approaches in peptidomics, where clustering reduces dataset dimensionality and improves downstream interpretability while preserving functionally related sequence space [39].

Representative peptides from each cluster were selected to probe potential interaction space. Given the central role of short peptide motifs in mediating protein interactions and the limited availability of experimentally resolved peptide–protein complexes, molecular docking provides a pragmatic strategy to explore potential binding landscapes in silico. For docking terminology, “receptor” refers to the screened protein structure and does not imply a biological receptor function.

Because no experimentally calibrated universal PRODIGY cutoff is available for heterogeneous peptide–protein screening, the GO analysis was evaluated across increasing receptor-ranking depths rather than by imposing an absolute predicted-energy threshold. The Jaccard procedure was used solely to identify the earliest local region in which the set of enriched GO terms became relatively insensitive to addition of lower-ranked candidate receptors. It was therefore treated as a ranking-depth selection heuristic rather than as independent evidence of biological validity.

Sensitivity analysis confirmed that the precise convergence depth depends to some extent on the operational definition. For Peptide 4, the reference criterion of *J* ≥ 0.80 for three consecutive valid transitions selected *N* = 185. Thresholds from 0.75 to 0.85 with the same three-transition window also selected *N* = 185, whereas requiring longer consecutive windows shifted the selected depth to later portions of the ranking. Thus, *N* = 185 represents the first local convergence point under the reference criterion rather than a unique or globally invariant biological cutoff. Complete ranking-depth trajectories and convergence-parameter sensitivity analyses are provided in Figure S1 and Table S3A–D. Under the reference criterion, convergence-selected ranking depths ranged from *N* = 85 to *N* = 315 across the 10 representative peptides, consistent with peptide-specific differences in the evolution of GO-enrichment profiles across the receptor rankings.

#### 3.3.1. Robustness of Representative-Peptide Selection

The submitted PCA–UMAP/GMM analysis produced 10 clusters with an overall silhouette score of 0.433, indicating moderate rather than complete separation of peptide groups. Sensitivity analyses confirmed that the exact partitioning of the 219 peptides was not invariant to analytical choices. When clustering was performed in PCA space without UMAP, the minimum-BIC solution contained eight clusters, and agreement with the submitted 10-cluster solution was modest (ARI = 0.241); constraining the PCA-only analysis to 10 clusters increased agreement to ARI = 0.411. Exclusion of predicted structural descriptors also altered the preferred number and membership of clusters. Across alternative UMAP random seeds, however, cluster assignments retained moderate reproducibility, with a mean ARI of 0.659, while resampling analyses yielded a mean ARI of 0.553. These results indicate genuine organization within the peptide feature space but do not support interpretation of the 10 clusters as uniquely defined or invariant biological classes.

We therefore evaluated the selected peptides as a diversity-sampling panel independently of cluster identity. Across 10,000 randomly generated 10-peptide panels, the observed representative set provided significantly greater coverage of the full peptide feature space. In the complete sequence–structure–physicochemical representation, the mean nearest-representative distance was 8.30% lower than expected for random 10-peptide panels (*P* = 2.0 × 10^−4^), with a 7.62% reduction in the median nearest-representative distance (*P* = 0.0059). Similar results were obtained after exclusion of predicted structural descriptors: mean and median nearest-representative distances were reduced by 9.57% (*P* = 6.0 × 10^−4^) and 10.89% (*P* = 8.0 × 10^−4^), respectively. Thus, although the exact cluster boundaries and medoid identities depend on the feature representation and dimensionality-reduction procedure, the selected 10-peptide panel samples the observed GAD peptide landscape more broadly than would be expected from random selection.

#### 3.3.2. Randomized-Affinity Negative Control

To determine whether comparable GO enrichment could arise from arbitrary receptor rankings, docking-derived affinity scores were randomized across protein identities and the enrichment analysis was repeated 500 times for each representative peptide at that peptide’s observed convergence-selected ranking depth. Significant GO enrichment was uncommon under randomization. Across the 10 representative peptides, only 0.4–2.2% of randomized rankings produced any FDR-significant GO Biological Process term, and the median and 95th-percentile numbers of significant terms were zero for every peptide. In contrast, the observed profiles contained between 2 and 72 significant terms. For nine of the 10 peptides, none of the 500 randomized rankings produced as many significant terms as observed, corresponding to an empirical *p* value of 1/501 = 0.001996. For Peptide176, three of 500 randomized rankings produced at least as many significant terms as the observed two-term profile, yielding an empirical *p* value of 4/501 = 0.007984.

For Peptide4 specifically, the original docking-derived ranking contained nine significant GO terms at the convergence-selected depth of N = 185. In the corresponding randomized null analysis, only 3 of 500 randomized rankings produced any significant GO enrichment, and no randomized ranking produced more than two significant terms. Thus:

$$P_{\mathrm{emp}}\;=\;\frac{1\;+\;0}{500\;+\;1}\;=\;\frac{1}{501}\;=\;0.001996$$

Taken together, all 10 representative peptides showed greater GO-enrichment magnitude than expected after random disruption of the score-to-protein relationship. These findings support the presence of non-random functional structure in the docking-derived receptor rankings, while not establishing the correctness of individual predicted peptide-protein interactions.

To evaluate the effect of selecting the most favorable of four docked poses, the complete pose-level output for Peptide 4 was reanalyzed. All 4960 peptide–receptor pairs contained four successfully scored poses, yielding 19,840 pose-level PRODIGY predictions. Pose-to-pose variability was measurable, with a median within-receptor standard deviation of 1.59 kcal/mol and a median score range of 3.53 kcal/mol. The median difference between the best pose-level estimate and the within-receptor median was 1.59 kcal/mol, indicating an expected optimistic shift associated with minimum-score selection.

Despite this shift in absolute score, the overall receptor ranking was highly preserved when alternative aggregation procedures were used. The best-of-four and median-of-four rankings showed a Spearman correlation of ρ = 0.9875 and Kendall τ = 0.9147, whereas the best-of-four and mean-of-four rankings showed ρ = 0.9912 and τ = 0.9282. At the original convergence-selected depth of N = 185, 142 of 185 receptors (76.8%) from the primary best-of-four target set were retained under median aggregation, and 148 of 185 (80.0%) were retained under mean aggregation. A detailed pose-aggregation sensitivity analysis performed for Peptide 4 is provided as an illustrative case in Table S3E.

Propagation of the alternative rankings through the GO enrichment workflow showed greater sensitivity in exact enrichment breadth and ranking-depth selection. The original best-of-four ranking reached the predefined Jaccard-based convergence criterion at N = 185, compared with N = 195 for median-of-four scoring and N = 375 for mean-of-four scoring. When each method was compared at its independently selected convergence depth, the top-GO-set Jaccard similarity was 0.364 between best and median aggregation and 0.097 between best and mean aggregation, reflecting both changes in ranking and differences in the number of proteins entering the enrichment analysis. At the common original depth of N = 185, however, median-of-four scoring retained 8 of the 9 GO terms identified in the primary analysis, while mean-of-four scoring retained 6 of the 9; all six terms identified under mean aggregation at this depth were also present in the original profile. These results indicate that exact target membership, enrichment breadth, and convergence depth are sensitive to pose-score aggregation, whereas the global receptor-ranking structure and a substantial portion of the core enrichment signal are preserved.

Because recurrence-based GO filtering could exclude strongly ranked peptide-specific candidates outside the recurrent functional categories, the GO-guided analysis was complemented by an independent review of the 10 most favorably ranked docking-derived PDB/chain candidates for each representative peptide. The combined procedure yielded 180 unique candidate protein targets, comprising 129 candidates identified through the GO-guided route and 51 additional candidates retained through the top-ranked docking route (Figure 5A). Candidate provenance, including the route of identification and associated peptide, PDB/chain, and GO information where applicable, is provided in Table S2. These provenance assignments permit the interpreted target set to be traced directly to the computational selection step rather than relying solely on post hoc biological classification. Candidate interactors were classified into five physiological domains based on their primary protein-centric function (Figure 5B).

Identified domains included Innate immune and inflammatory defense, Protease activity and peptide processing, Extracellular matrix remodeling, Tissue repair and Wound Healing, Apoptotic Signaling and Epithelial Turnover. Selected hypothesis-generating candidate interactor families and their association with potential upper GI biological context for further investigation and potential physiological relevance are presented in Table 1. The full list of potential interactors is presented in Table S2.

To quantify potential representation bias in the experimentally structure-accessible receptor library, screened proteins were mapped to unique human UniProt accessions and compared with the reviewed human reference proteome (UniProt release 2026_03). Of 4392 unique human UniProt accessions represented in the docking library, 4378 (99.68%) were present in the 20,431-protein reviewed reference set, corresponding to approximately 21.4% of reviewed human proteins. Significant differences in representation were observed across broad cellular localization, GO Cellular Component, protein-family, and functional annotation categories after FDR correction. The screened library was enriched for several well-represented structural classes, including cytoplasmic, nuclear, mitochondrial, and kinase-associated proteins, whereas selected classes, including G-protein-coupled receptors and olfactory receptors, were underrepresented. Screened proteins also had substantially higher annotation coverage than the unscreened reference proteome across subcellular-location and GO annotation dimensions. These results demonstrate that the experimentally structure-accessible screening universe is not an unbiased representation of the human proteome and should be interpreted as a structure-access-constrained search space. Detailed per-protein annotations and statistical comparisons of annotation coverage, cellular localization, protein-family representation, GO Cellular Component, GO Molecular Function, and GO Biological Process categories are provided in Table S4.

## 4. Discussion

The present study identified 219 soy-derived peptides in GAD and revealed clear descriptive differences between the peptide profiles of GAD and unfermented soy flour. The identified GAD peptides contained numerous overlapping sequence families and N- and C-terminal truncation patterns. Three literature-reported antioxidant peptides, VNPESQQGSPR, LVPPQESQ, and EITPEKNPQ, were identified as exact contiguous sequences within longer GAD peptides. This observation provides sequence-level precedent for investigating the peptide fraction but does not establish that the longer peptides share the activity of the corresponding free sequences or that the embedded peptides are released under physiological conditions. Moreover, identification of only a limited number of previously characterized sequences through literature comparison illustrates the limitations of relying exclusively on known peptide annotations. The broader computational workflow was therefore used to explore and prioritize potential biological contexts across a chemically diverse peptide mixture for which most individual sequences have not been characterized.

Ten representative peptides were selected to sample the observed peptide-feature diversity and were used for computational docking, which generated ranked lists of candidate human protein interactors. Subsequent prioritization based on computational docking scores, upper-GI relevance, tissue and subcellular localization, and plausible peptide accessibility organized these candidates into broad biological contexts, including luminal proteolysis and peptide processing, mucosal immune and inflammatory regulation, mucosal barrier and extracellular-matrix remodeling, epithelial injury and turnover, and epithelial restitution and tissue repair. Collectively, these contexts identify mucosal integrity, injury responses, and tissue restitution as priority areas for future investigation of the GAD peptide fraction. These areas are also relevant to the gastric-injury findings reported in rat and equine models using the complete formulation. Future studies should determine whether the peptide fraction contributes to these product-level effects and validate the occurrence and functional consequences of selected candidate peptide-protein interactions.

The effects of GAD have been evaluated in individuals with diverse upper-gastrointestinal symptoms [11,12,14], including heartburn, acid reflux, bloating, belching, and abdominal pain, which may occur in functional dyspepsia or GERD [13]. Across these studies, improvements in symptom scores and quality-of-life measures support a role for this fermented postbiotic in promoting gastric comfort. However, the biological contexts emerging from the present candidate-interactor analysis appear more closely aligned with the earlier Gastropharm studies in individuals with gastritis or gastric and duodenal ulcers [8,9,10] and with preclinical findings in gastric-injury and ulcer models [15,16]. This pattern does not exclude the possibility that GAD peptides could contribute to physiological processes relevant to heartburn, reflux-related discomfort, or other gastric-comfort outcomes. Rather, it may partly reflect an inherent feature of the prioritization framework, which emphasized physiologically plausible peptide accessibility. The principal biological interpretation was restricted to Tier 1 and Tier 2 candidates with direct or context-dependent extracellular accessibility, whereas intracellular and otherwise less accessible Tier 3 candidates were retained as exploratory predictions in Table S2. Thus, Tier 2 candidates are those that become extracellularly accessible primarily through inflammatory-cell recruitment, secretion, proteolytic activation, plasma extravasation, or mucosal injury, thereby directing the prioritized landscape toward mucosal defense, injury responses, extracellular-matrix remodeling, and tissue restitution.

In addition, GO enrichment results are influenced by annotation density and database structure, which may favor well-characterized biological processes. To assess whether the observed enrichment magnitude could nevertheless arise from arbitrary docking-score assignments, 500 randomized-affinity controls were performed independently for each representative peptide while preserving the empirical score distribution and observed ranking depth. Comparable enrichment was rare under randomization: empirical permutation *p* values based on the number of FDR-significant GO terms were ≤0.008 for all 10 representative peptides. These controls support the conclusion that the observed GO profiles contain non-random structure associated with the docking-derived receptor rankings. They do not, however, demonstrate that individual peptide-protein interactions are experimentally correct, and the docking results should therefore remain interpreted as a prioritization layer for hypothesis generation.

Several limitations should also be acknowledged. Peptide sequences were identified by MS/MS in pooled samples and subsequently annotated in the individual-lot MS1 datasets using the predefined mass and migration-time tolerances. The individual-lot signals therefore indicate detection of corresponding MS1 features rather than independent MS/MS confirmation of each sequence in every manufacturing lot. Nevertheless, Table S1 provides the relative signals and detection status across the two soy-flour and four GAD lots, while PCA provides a descriptive assessment of overall profile consistency and lot-to-lot variation. In addition, reducing the 219 identified peptides to a 10-peptide panel necessarily limits the interaction space explored by the proteome-scale docking analysis. Although the selected representatives provided significantly broader coverage of the observed peptide feature space than random 10-peptide panels, exact cluster assignments and medoid identities showed moderate sensitivity to feature representation, dimensionality reduction, and resampling. The selected peptides should therefore be interpreted as a diversity-oriented sampling panel rather than as definitive biological proxies for all peptides within their assigned clusters. Consequently, motif-specific or low-frequency interaction phenotypes not captured by the clustering representation may have been missed.

The use of predicted structures for short peptides introduces conformational uncertainty into both feature construction and downstream docking. Peptides in this study ranged from 6 to 45 amino acids, and sequences of this length may be conformationally flexible or intrinsically disordered in solution and may adopt receptor-dependent conformations upon binding. Accordingly, a single OpenFold3-predicted free-peptide structure should not be interpreted as a unique native conformation or as a complete representation of the accessible conformational ensemble. Although representative-peptide selection remained supported when predicted structural descriptors were excluded from the clustering representation, alternative peptide conformations were not propagated through the structure-library-scale docking workflow. Individual docking poses and target rankings may therefore depend in part on the starting peptide conformation, and future target-specific studies should evaluate conformational ensembles or experimentally informed peptide structures where appropriate. In addition, the primary analysis retained the most favorable PRODIGY-predicted binding free-energy estimate from the four generated poses. The Peptide 4 sensitivity analysis demonstrated that this choice produces an expected optimistic shift in absolute predicted score, although the resulting structure-library-scale docking was highly correlated with rankings based on median- or mean-of-four aggregation. Individual target membership and the precise GO ranking depth were more sensitive to aggregation strategy, indicating that fine differences among individual predicted targets should not be overinterpreted.

Receptor preparation introduces an additional source of uncertainty. Bound ligands, crystallographic waters, ions, and cofactors were removed uniformly before docking to provide a standardized input representation across the approximately 5000-protein library. This simplification may alter binding-site geometry, electrostatics, or accessibility for proteins that depend on catalytic metals, prosthetic groups, or structurally important waters and cofactors. Consequently, predictions involving such proteins should not be interpreted as reconstructions of their native holo-state interactions. Target-specific follow-up calculations retaining biologically essential structural components would be appropriate for prioritized proteins.

More broadly, reliance on experimentally determined structures restricts the screen to a subset of the human proteome. Quantitative comparison with the reviewed human reference proteome demonstrated that this experimentally structure-accessible protein universe is biased with respect to protein class, cellular and subcellular localization, functional annotation, and annotation density. Accordingly, absence of a protein from the screened receptor library should not be interpreted as evidence of absence of biological interaction. GO enrichment also remains influenced by annotation density and ontology structure. The docking scores used here should therefore be interpreted as screening-level predicted binding-energy estimates for relative target prioritization rather than as experimentally calibrated binding affinities or confirmation of individual peptide–protein interactions. The principal value of the analysis is consequently in identifying reproducible ranking patterns and systems-level biological hypotheses that can guide subsequent target-specific structural and experimental validation.

The Jaccard-based convergence procedure should likewise be interpreted as a data-reduction heuristic rather than a validation metric. It identifies a local region of the ranked receptor list in which the enriched GO-term composition becomes comparatively insensitive to the addition of lower-ranked candidates, but it does not establish that the selected ranking depth is biologically optimal or that the underlying peptide–protein interactions are correct. Sensitivity to the Jaccard threshold, convergence window, and pose-score aggregation further indicates that the exact selected depth should not be overinterpreted.

To address these limitations, the present study incorporated clustering sensitivity analyses, randomized-affinity controls, pose-aggregation analyses, and recurrence across independently derived GO profiles, while emphasizing broad biological contexts rather than individual target predictions. The molecular complexity of GAD supports a multi-component model in which several peptide families and other fermentation-derived constituents may contribute to the biological properties of the complete formulation. The relative contributions of these components, and whether their activities are additive, complementary, or interactive, remain to be determined.

## 5. Conclusions

Overall, this study provides a systems-oriented framework for investigating complex peptide mixtures by integrating peptidomic characterization, computational interaction modeling, and candidate-target prioritization based on biological context. The approach linked a diversity-sampling peptide panel with candidate interactors, yielding testable biological contexts rather than attributing activity to a single peptide or molecular target. The prioritized themes, particularly mucosal immune regulation, extracellular-matrix remodeling, epithelial turnover, and tissue restitution, provide a biologically informed basis for future mechanistic studies of this multifunctional fermented postbiotic.

## Figures and Tables

**Figure 1:**
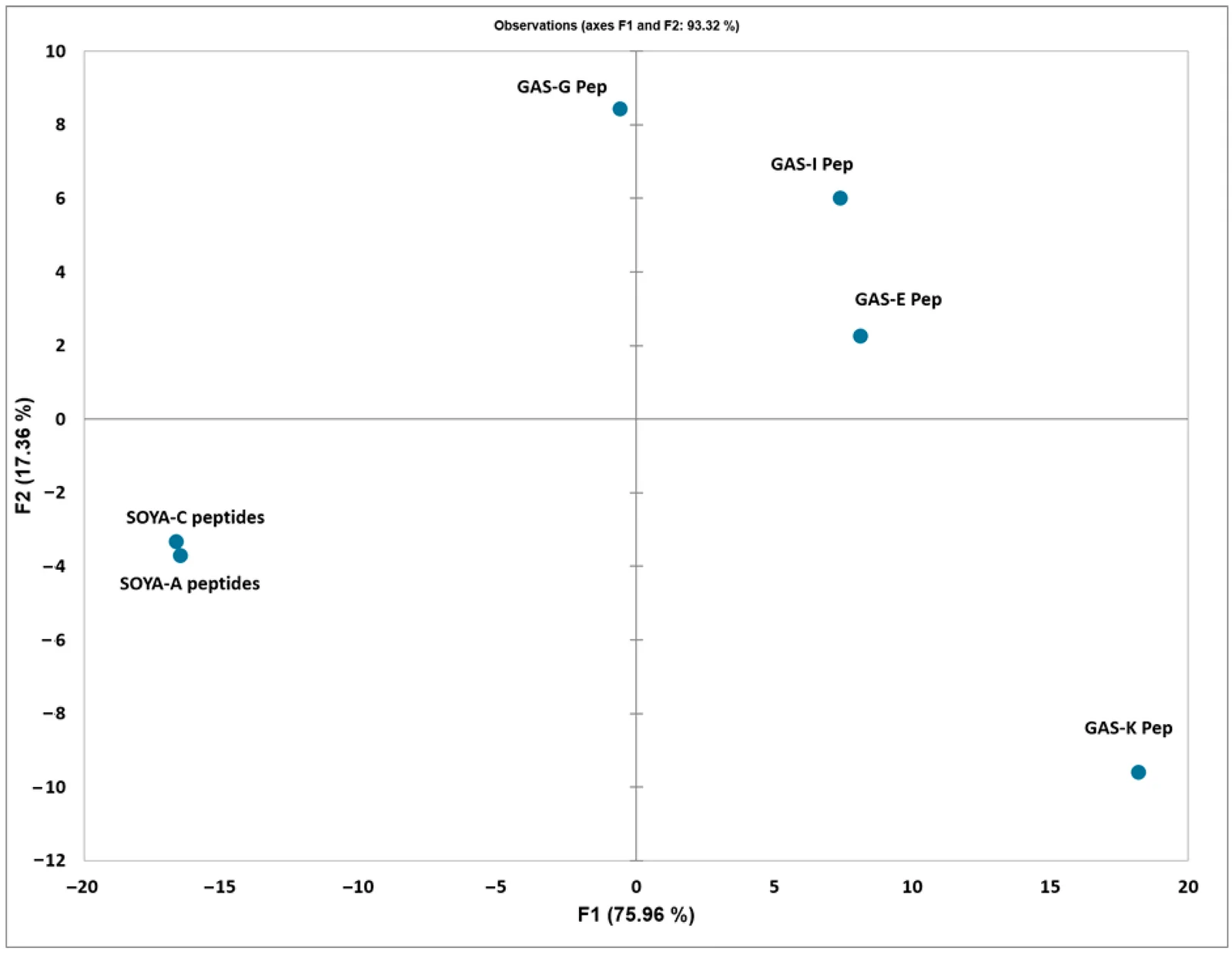
Principal component analysis of the identified peptide profiles in soy flour and GAD. PCA was performed using the relative signal profiles of the 219 identified peptides. Each point represents an independent manufacturing lot. F1 and F2 accounted for 75.96% and 17.36% of the total variance, respectively, and together, they explained 93.32% of the observed variance. The analysis was used as an exploratory visualization of overall peptide-profile variation.

**Figure 2:**
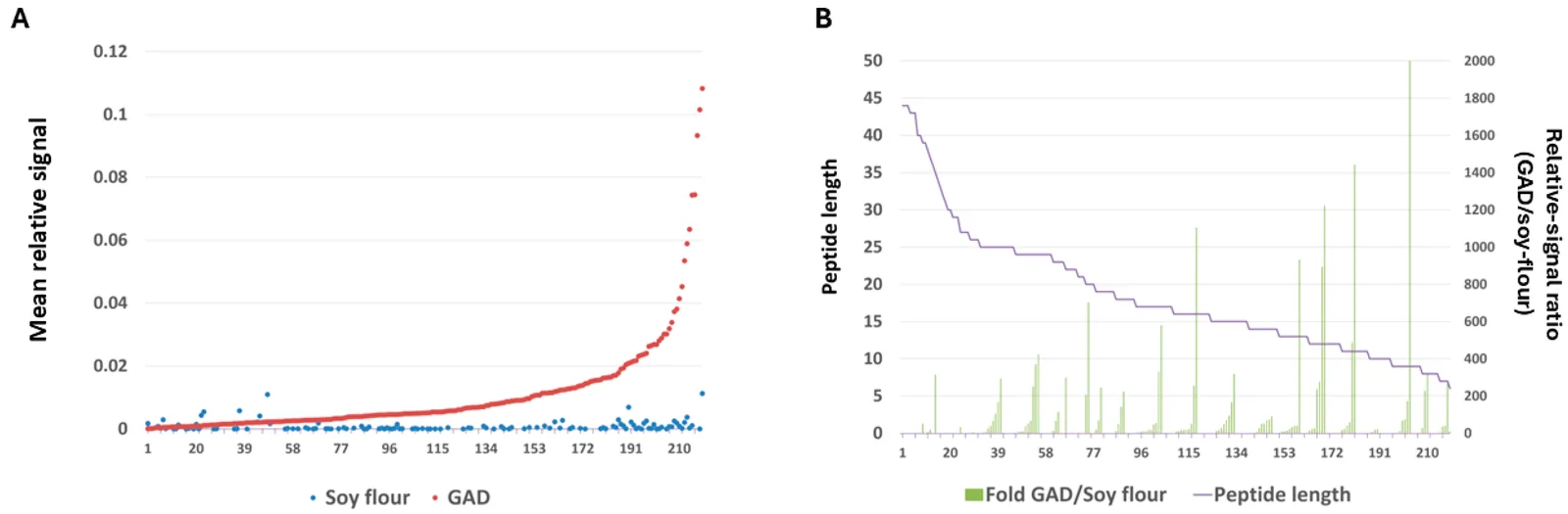
Descriptive distribution of peptide relative signals and GAD-to-soy-flour relative-signal ratios. (**A**) Mean relative signals of the 219 identified peptides in soy flour (blue) and GAD (red), ordered by increasing mean relative signal in GAD. A subset of peptides showed markedly higher mean relative signals in GAD than in soy flour. (**B**) Peptide length (purple) and GAD-to-soy-flour relative-signal ratio (green). GAD-to-soy-flour ratios were calculated by dividing the mean relative signal in GAD by the mean relative signal in soy flour. High ratios reflect peptide features with very low relative signals in soy flour and should be interpreted descriptively. Numerical ratios were not calculated when the peptide was not detected in both soy-flour lots. No peptide-level inferential statistical testing was performed.

**Figure 3:**
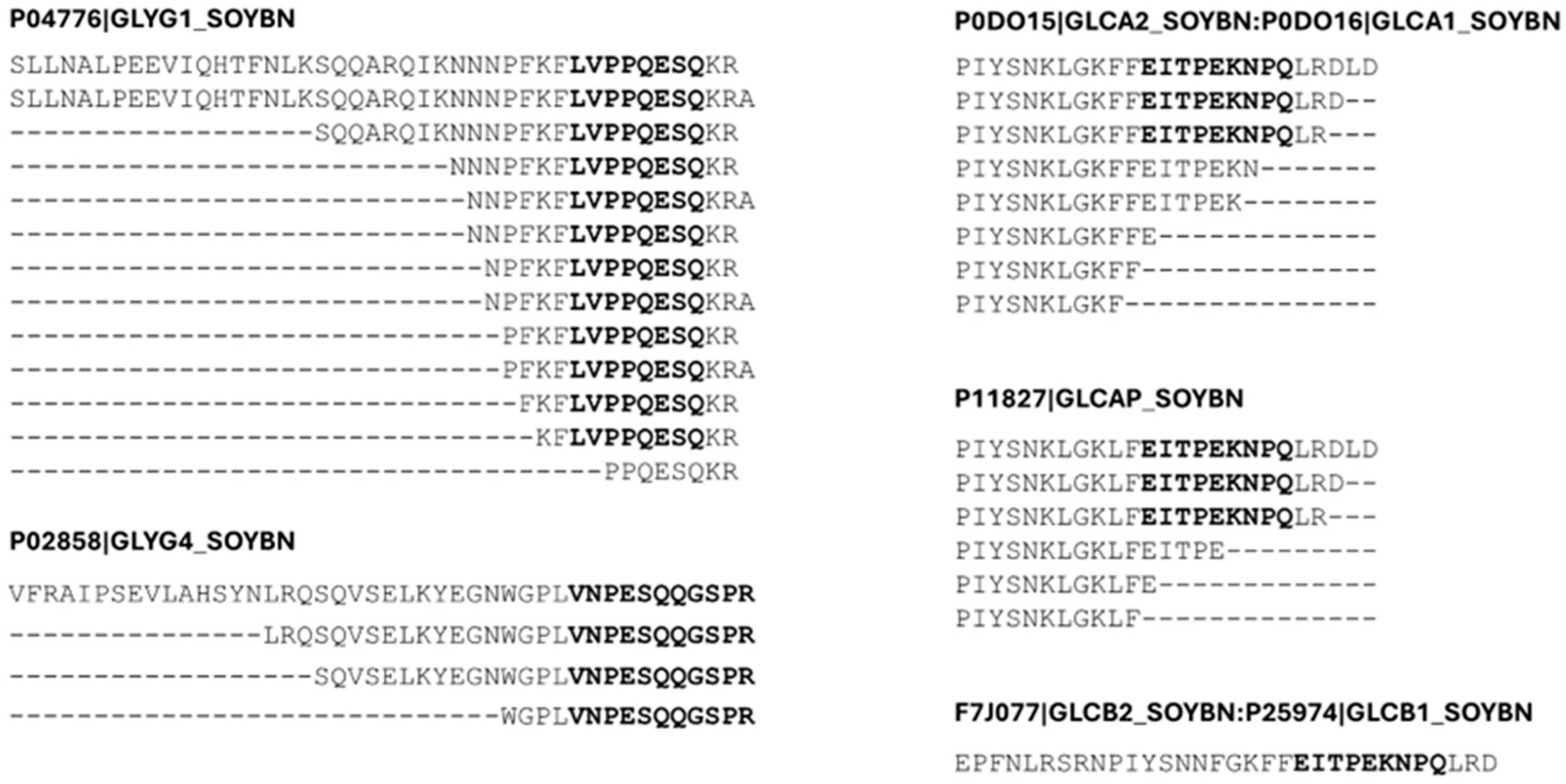
Literature-reported antioxidant peptide sequences embedded within longer GAD peptides. The sequences VNPESQQGSPR, LVPPQESQ, and EITPEKNPQ are present as exact contiguous sequences within the indicated longer GAD peptides, representing 100% sequence identity over the complete length of each literature peptide. Additional N- and/or C-terminal residues belonging to the longer GAD sequences are shown outside the highlighted regions. EITPEKNPQ was identified within peptides mapped to three soybean source proteins. Antioxidant activity was previously demonstrated for the exact shorter peptides (in bold) but not for the longer GAD peptides shown here. The presence of an embedded sequence does not establish its release, cellular uptake, potency, or biological activity within the longer peptide context.

**Figure 4:**
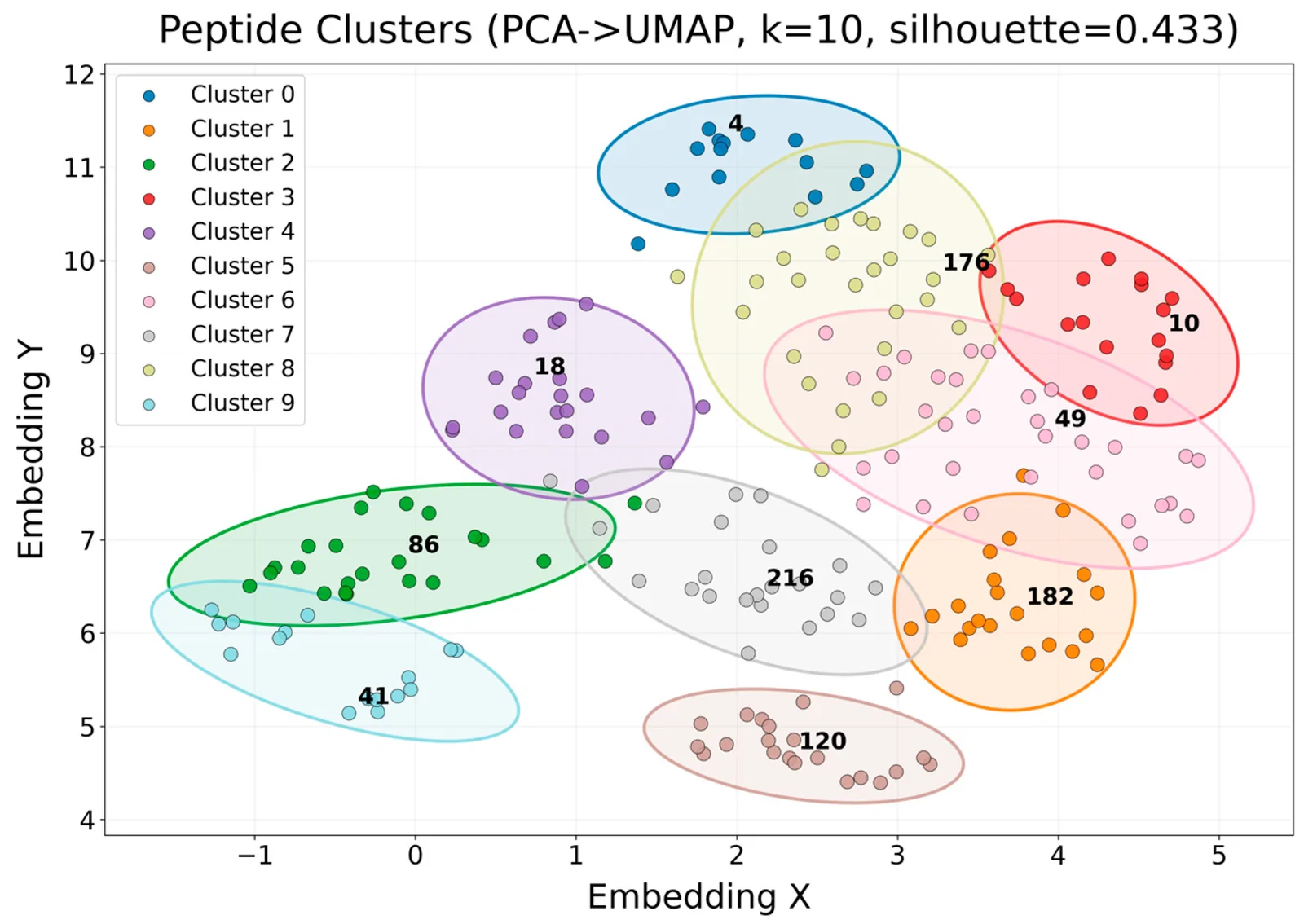
Physicochemical clustering of GAD peptides and selection of representative peptide families. GAD peptides were clustered in the integrated peptide-feature space using PCA–UMAP embedding and Gaussian-mixture modeling (k = 10; silhouette score = 0.433). Each point represents a peptide, with colors indicating cluster membership. Labeled points correspond to the representative peptides selected for docking and interactor analyses. The labeled peptides were selected as diversity-sampling representatives spanning distinct regions of the observed peptide feature space. Because cluster membership showed moderate sensitivity to dimensionality reduction and feature selection, these representatives are used as a tractable sampling panel for downstream docking rather than as definitive proxies for all biological activities of peptides within their respective clusters. Cluster sizes were as follows: cluster 0, n = 13; cluster 1, n = 21; cluster 2, n = 23; cluster 3, n = 18; cluster 4, n = 20; cluster 5, n = 22; cluster 6, n = 34; cluster 7, n = 23; cluster 8, n = 28; and cluster 9, n = 17. The exact sequence of the peptides is included in Table S1.

**Figure 5:**
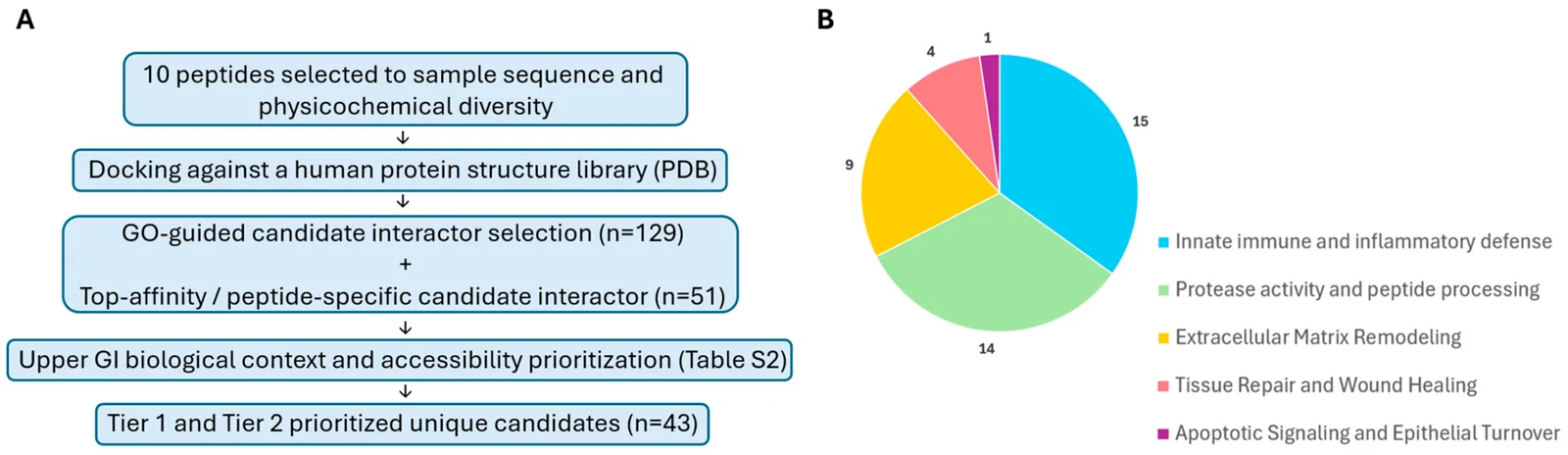
Selection and functional classification of prioritized candidate interactors. (**A**) Schematic of the computational selection and prioritization workflow. (**B**) Functional distribution of the 43 unique Tier 1 and Tier 2 candidates retained through the GO-guided and peptide-specific top-ranked docking routes. Each biological target was counted once and assigned to one primary protein-centric functional domain.

**Table 1:** Potential biological contexts and physiological relevance emerging from Tier 1 and Tier 2 candidate interactors.

Potential Upper-GI biological Context for Future Investigation ^1^	Representative Candidate Families or Candidate Interactors	General Physiological Relevance to Investigate
Luminal proteolysis and peptide processing	Uropepsin/pepsin A; kallikreins KLK1, KLK5, KLK6, KLK7, and KLK8; cathepsins E and G; matriptase; prostasin; enteropeptidase; chymotrypsin C; carboxypeptidase O; mesotrypsin/PRSS3	Digestion and processing of proteins and peptides; regulation of extracellular proteolytic cascades and the local gastric peptide environment.
Mucosal immune activation and inflammatory regulation	Granzymes A, B, H, M, and pro-granzyme K; NSP4; neutrophil elastase; azurocidin/CAP37; MASP-1 and MASP-2; C1s; IL-17RA; IL-22RA1; Mincle/CLEC4E; VISTA	Immune-cell recruitment and degranulation; complement activation; microbial and damage recognition; inflammatory signaling and its eventual resolution.
Mucosal barrier integrity and extracellular-matrix remodeling	MMP family, including proMMP-1, proMMP-9, proMMP-10, MMP-7, MMP-8, and MMP-13; neutrophil elastase; ADAMTS-5; ED-B fibronectin; uPARAP/Endo180; matriptase and prostasin	Barrier and basement-membrane turnover; removal of damaged matrix; epithelial permeability; mucus-associated defense; restructuring of injured mucosa.
Epithelial injury, apoptosis, and cell turnover	TRAIL-R2; granzymes A, B, H, M, and pro-granzyme K; NSP4; neutrophil elastase; IL-17RA; IL-22RA1	Regulated removal of damaged epithelial cells; inflammation-associated epithelial loss; cell turnover preceding mucosal restoration.
Epithelial restitution and tissue repair	HGFA; uPA; thrombospondin-1; ED-B fibronectin; uPARAP/Endo180; ADAMTS-5; MMP family, including proMMP-1, proMMP-9, proMMP-10, MMP-7, MMP-8, and MMP-13	Epithelial migration and re-epithelialization; angiogenesis; matrix organization; mucus and barrier restoration; wound closure.

^1^ Biological contexts are non-exclusive because inflammatory regulation, extracellular-matrix remodeling, epithelial turnover, and tissue repair are interconnected processes. The listed contexts identify priority areas for future investigation of GAD peptide activity. Because docking does not establish the occurrence, direction, or functional consequence of an interaction, experimental studies are required to determine whether and how individual peptides modulate these targets and associated biological processes.

## Data Availability

The data presented in this study are included in the article/Supplementary Materials.

## Supplementary Materials

The following supporting information can be downloaded at: https://www.mdpi.com/article/10.3390/metabo16100794/s1, Table S1: Complete peptidomics dataset for soy flour and GAD; Table S2: Candidate protein interactors dataset and tier justification; Supplementary Methods, Figure S1, and Table S3A–E: GO ranking-depth convergence and sensitivity analyses; Table S4: Annotation coverage and representativeness of the experimentally structure-accessible human protein library relative to the reviewed human proteome.

## Abbreviations

The following abbreviations are used in this manuscript:

CE-FTMS	Capillary electrophoresis–Fourier transform mass spectrometry
GAD	Gastro-AD
GO	Gene Ontology
KEAP1	Kelch-like ECH-associated protein 1
MS/MS	Tandem mass spectrometry
NMR	Nuclear magnetic resonance
NRF2	Nuclear factor erythroid 2-related factor 2
PDB	Protein Data Bank
