# Supplementary material for: Peptidomics and Computational Docking Prioritize Candidate Peptide–Protein Associations Relevant to Upper-Gastrointestinal Biology in a Fermented Soy-Derived Postbiotic
Source: Metabolites. 2026 Oct 19;16(10):794. doi: 10.3390/metabo16100794 (PMC13707153; doi:10.3390/metabo16100794)
Supplement: Supplementary file 1 [file metabolites-16-00794-s001.zip › Supplementary file_GO ranking-depth convergence_Table S3A-E and Figure S1.pdf]

Supplementary Methods: GO ranking-depth convergence

For each representative peptide, GO Biological Process enrichment was evaluated across increasing receptor-ranking depths in 10-row increments. At each valid ranking depth, significant GO Biological Process terms were ranked by fold enrichment and the top 25 terms, or all significant terms when fewer than 25 were available, were retained. Jaccard similarity was calculated only between successive valid ranking depths containing at least one significant GO term. The reference convergence criterion was J >= 0.80 for three consecutive valid transitions, and the earliest ranking depth satisfying this rule was selected for downstream interpretation. The criterion was used as a ranking-depth selection heuristic and not as evidence that the selected depth was biologically optimal or that individual docking predictions were correct.

Jaccard coefficient: J(A,B) = |A intersection B| / |A union B|, where A and B are the retained significant GO-term sets at successive valid ranking depths.

Trajectory reporting: The archived convergence runs used early stopping once the first qualifying three-transition region was detected. Accordingly, for nine peptides the tables below report every valid transition actually evaluated through the selected depth. Peptide4 was subsequently subjected to a corrected complete reviewer-validation sweep through N = 505, which is reported in full and used as the authoritative Peptide4 trajectory in the revised manuscript.

Sensitivity analysis: For Peptide4, the convergence definition was varied across Jaccard thresholds of 0.70, 0.75, 0.80, 0.85, and 0.90 and persistence windows of 2, 3, 4, or 5 consecutive valid transitions.

Supplementary Table S3A. Observed convergence-selection summary across representative peptides

Representative peptide	First valid N	Convergence-selected N	Last evaluated N	Jaccard transitions evaluated	Early stopping
Peptide4	85	185	505	42	No (full sweep to 505)
Peptide10	25	185	185	16	Yes (at first qualifying region)
Peptide18	135	195	195	6	Yes (at first qualifying region)
Peptide41	125	285	285	16	Yes (at first qualifying region)
Peptide49	55	165	165	11	Yes (at first qualifying region)
Peptide86	35	315	315	28	Yes (at first qualifying region)
Peptide120	45	85	85	4	Yes (at first qualifying region)
Peptide176	65	145	145	8	Yes (at first qualifying region)
Peptide182	35	255	255	22	Yes (at first qualifying region)
Peptide216	25	95	95	7	Yes (at first qualifying region)

Note. The reference criterion was J >= 0.80 for three consecutive valid transitions. Peptide4 uses the corrected full reviewer-validation trajectory (selected N = 185). The archived Peptide4 stability report supplied separately selected N = 265 and is not used here because it conflicts with the corrected trajectory and the N = 185 analysis used in the revised manuscript and permutation controls.

Supplementary Figure S1A. Corrected complete Peptide4 Jaccard trajectory

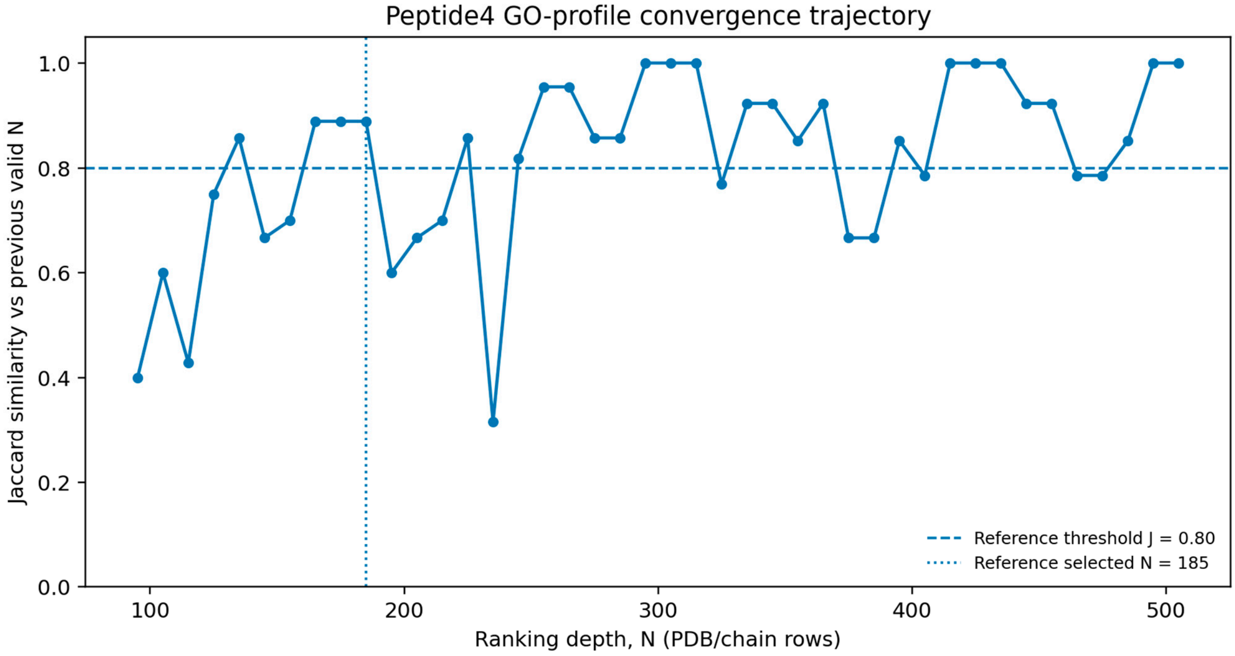

Figure S1A. Jaccard similarity between successive valid GO-enrichment profiles for Peptide4 across the corrected complete N = 25-505 ranking-depth sweep. Ranking depths with no significant GO terms were excluded from consecutive Jaccard calculations. The horizontal reference line denotes J = 0.80; the vertical reference line marks N = 185, the first depth satisfying three consecutive valid transitions at or above the reference threshold.

Supplementary Figure S1B. Peptide4 convergence-definition sensitivity

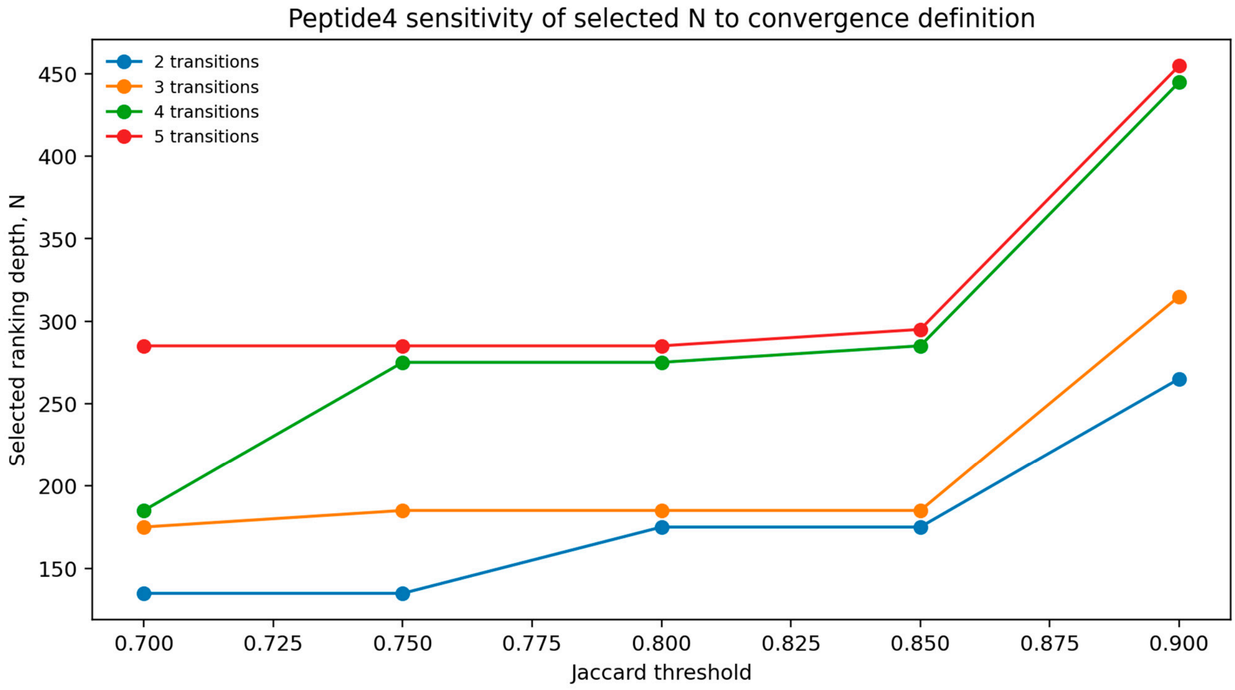

Figure S1B. Sensitivity of the convergence-selected Peptide4 ranking depth to the Jaccard threshold and number of consecutive valid transitions required. The reference definition (J >= 0.80 for three transitions) selected N = 185.

Supplementary Table S3B. Jaccard trajectories for all 10 representative peptides

Peptide	Previous valid N	Current valid N	Jaccard similarity	Reference threshold met?	Current N is selected depth?
Peptide4	85	95	0.4000	No	
Peptide4	95	105	0.6000	No	
Peptide4	105	115	0.4286	No	
Peptide4	115	125	0.7500	No	
Peptide4	125	135	0.8571	Yes	
Peptide4	135	145	0.6667	No	
Peptide4	145	155	0.7000	No	
Peptide4	155	165	0.8889	Yes	
Peptide4	165	175	0.8889	Yes	
Peptide4	175	185	0.8889	Yes	Yes
Peptide4	185	195	0.6000	No	
Peptide4	195	205	0.6667	No	
Peptide4	205	215	0.7000	No	
Peptide4	215	225	0.8571	Yes	
Peptide4	225	235	0.3158	No	
Peptide4	235	245	0.8182	Yes	
Peptide4	245	255	0.9545	Yes	
Peptide4	255	265	0.9545	Yes	
Peptide4	265	275	0.8571	Yes	
Peptide4	275	285	0.8571	Yes	
Peptide4	285	295	1.0000	Yes	
Peptide4	295	305	1.0000	Yes	
Peptide4	305	315	1.0000	Yes	
Peptide4	315	325	0.7692	No	
Peptide4	325	335	0.9231	Yes	
Peptide4	335	345	0.9231	Yes	
Peptide4	345	355	0.8519	Yes	
Peptide4	355	365	0.9231	Yes	
Peptide4	365	375	0.6667	No	
Peptide4	375	385	0.6667	No	
Peptide4	385	395	0.8519	Yes	
Peptide4	395	405	0.7857	No	
Peptide4	405	415	1.0000	Yes	
Peptide4	415	425	1.0000	Yes	
Peptide4	425	435	1.0000	Yes	
Peptide4	435	445	0.9231	Yes	
Peptide4	445	455	0.9231	Yes	
Peptide4	455	465	0.7857	No	
Peptide4	465	475	0.7857	No	
Peptide4	475	485	0.8519	Yes	
Peptide4	485	495	1.0000	Yes	

Peptide	Previous valid N	Current valid N	Jaccard similarity	Reference threshold met?	Current N is selected depth?
Peptide4	495	505	1.0000	Yes	
Peptide10	25	35	0.5000	No	
Peptide10	35	45	1.0000	Yes	
Peptide10	45	55	0.6667	No	
Peptide10	55	65	1.0000	Yes	
Peptide10	65	75	1.0000	Yes	
Peptide10	75	85	0.6000	No	
Peptide10	85	95	0.6250	No	
Peptide10	95	105	0.5000	No	
Peptide10	105	115	0.6667	No	
Peptide10	115	125	0.1923	No	
Peptide10	125	135	0.7857	No	
Peptide10	135	145	0.4286	No	
Peptide10	145	155	0.6667	No	
Peptide10	155	165	0.9231	Yes	
Peptide10	165	175	1.0000	Yes	
Peptide10	175	185	1.0000	Yes	Yes
Peptide18	135	145	0.5000	No	
Peptide18	145	155	0.3333	No	
Peptide18	155	165	0.5714	No	
Peptide18	165	175	1.0000	Yes	
Peptide18	175	185	0.8333	Yes	
Peptide18	185	195	0.8571	Yes	Yes
Peptide41	125	135	0.3529	No	
Peptide41	135	145	0.5000	No	
Peptide41	145	155	0.7241	No	
Peptide41	155	165	0.8519	Yes	
Peptide41	165	175	0.6667	No	
Peptide41	175	185	0.5625	No	
Peptide41	185	195	0.7241	No	
Peptide41	195	205	0.6667	No	
Peptide41	205	215	0.8519	Yes	
Peptide41	215	225	0.7857	No	
Peptide41	225	235	0.8519	Yes	
Peptide41	235	245	0.7241	No	
Peptide41	245	255	0.7857	No	
Peptide41	255	265	0.8519	Yes	
Peptide41	265	275	0.8519	Yes	
Peptide41	275	285	1.0000	Yes	Yes
Peptide49	55	65	0.5000	No	
Peptide49	65	75	1.0000	Yes	
Peptide49	75	85	0.6667	No	
Peptide49	85	95	0.3750	No	
Peptide49	95	105	0.6250	No	

Supplementary Jaccard ranking-depth convergence analyses

Peptide	Previous valid N	Current valid N	Jaccard similarity	Reference threshold met?	Current N is selected depth?
Peptide49	105	115	0.8333	Yes	
Peptide49	115	125	0.7500	No	
Peptide49	125	135	0.7500	No	
Peptide49	135	145	1.0000	Yes	
Peptide49	145	155	0.8571	Yes	
Peptide49	155	165	0.8571	Yes	Yes
Peptide86	35	45	0.5000	No	
Peptide86	45	55	0.5000	No	
Peptide86	55	65	0.5000	No	
Peptide86	65	75	1.0000	Yes	
Peptide86	75	85	0.7500	No	
Peptide86	85	95	0.3333	No	
Peptide86	95	105	0.1429	No	
Peptide86	105	115	0.6667	No	
Peptide86	115	125	0.8889	Yes	
Peptide86	125	135	0.7778	No	
Peptide86	135	145	0.8750	Yes	
Peptide86	145	155	0.8889	Yes	
Peptide86	155	165	0.7500	No	
Peptide86	165	175	0.7500	No	
Peptide86	175	185	0.5185	No	
Peptide86	185	195	0.6667	No	
Peptide86	195	205	0.9231	Yes	
Peptide86	205	215	0.8519	Yes	
Peptide86	215	225	0.7857	No	
Peptide86	225	235	1.0000	Yes	
Peptide86	235	245	0.6129	No	
Peptide86	245	255	0.9231	Yes	
Peptide86	255	265	0.7857	No	
Peptide86	265	275	0.8846	Yes	
Peptide86	275	285	0.6250	No	
Peptide86	285	295	0.8333	Yes	
Peptide86	295	305	0.8889	Yes	
Peptide86	305	315	0.8333	Yes	Yes
Peptide120	45	55	0.3333	No	
Peptide120	55	65	1.0000	Yes	
Peptide120	65	75	1.0000	Yes	
Peptide120	75	85	1.0000	Yes	Yes
Peptide176	65	75	1.0000	Yes	
Peptide176	75	85	1.0000	Yes	
Peptide176	85	95	0.0000	No	
Peptide176	95	105	1.0000	Yes	
Peptide176	105	115	0.6667	No	
Peptide176	115	125	1.0000	Yes	

Peptide	Previous valid N	Current valid N	Jaccard similarity	Reference threshold met?	Current N is selected depth?
Peptide176	125	135	1.0000	Yes	
Peptide176	135	145	1.0000	Yes	Yes
Peptide182	35	45	0.5000	No	
Peptide182	45	55	0.6250	No	
Peptide182	55	65	0.3684	No	
Peptide182	65	75	0.5714	No	
Peptide182	75	85	0.6667	No	
Peptide182	85	95	0.4706	No	
Peptide182	95	105	0.6129	No	
Peptide182	105	115	0.9231	Yes	
Peptide182	115	125	0.6129	No	
Peptide182	125	135	0.8519	Yes	
Peptide182	135	145	0.8519	Yes	
Peptide182	145	155	0.7857	No	
Peptide182	155	165	0.7857	No	
Peptide182	165	175	0.8519	Yes	
Peptide182	175	185	0.7857	No	
Peptide182	185	195	0.7241	No	
Peptide182	195	205	0.9231	Yes	
Peptide182	205	215	0.6129	No	
Peptide182	215	225	0.7857	No	
Peptide182	225	235	0.8519	Yes	
Peptide182	235	245	0.8519	Yes	
Peptide182	245	255	0.9231	Yes	Yes
Peptide216	25	35	0.1429	No	
Peptide216	35	45	0.8750	Yes	
Peptide216	45	55	0.6364	No	
Peptide216	55	65	0.7000	No	
Peptide216	65	75	1.0000	Yes	
Peptide216	75	85	1.0000	Yes	
Peptide216	85	95	1.0000	Yes	Yes

Note. Rows shaded in light green mark the selected depth for each peptide. For Peptide4, the complete corrected reviewer-validation trajectory is shown through N = 505. For the other nine peptides, the archived analysis stopped once the first qualifying convergence region was detected, so no post-selection Jaccard values were generated in those runs.

Supplementary Table S3C. Peptide4 convergence-parameter sensitivity

Jaccard threshold	Consecutive valid transitions	Selected N	Top GO terms at selected N	Jaccard vs reference N=185 profile
0.70	2	135	6	0.667
0.70	3	175	8	0.889
0.70	4	185	9	1.000
0.70	5	285	21	0.304
0.75	2	135	6	0.667
0.75	3	185	9	1.000
0.75	4	275	18	0.350
0.75	5	285	21	0.304
0.80	2	175	8	0.889

Jaccard threshold	Consecutive valid transitions	Selected N	Top GO terms at selected N	Jaccard vs reference N=185 profile
0.80	3	185	9	1.000
0.80	4	275	18	0.350
0.80	5	285	21	0.304
0.85	2	175	8	0.889
0.85	3	185	9	1.000
0.85	4	285	21	0.304
0.85	5	295	21	0.304
0.90	2	265	21	0.304
0.90	3	315	21	0.304
0.90	4	445	25	0.062
0.90	5	455	25	0.062

Note. The highlighted row is the reference criterion. With a three-transition window, thresholds of 0.75, 0.80, and 0.85 all selected N = 185. Longer windows and/or a 0.90 threshold shifted the first qualifying convergence region to later ranking depths.

Supplementary Table S3D. Corrected complete Peptide4 ranking-depth trajectory

N	Unique genes	FDR-significant GO terms	Terms used for Jaccard	Valid enrichment	Previous valid N	Jaccard vs previous valid N
25	23	0	0	No		
35	33	0	0	No		
45	43	0	0	No		
55	53	0	0	No		
65	63	0	0	No		
75	71	0	0	No		
85	81	2	2	Yes		
95	90	5	5	Yes	85	0.400
105	99	3	3	Yes	95	0.600
115	109	7	7	Yes	105	0.429
125	119	7	7	Yes	115	0.750
135	128	6	6	Yes	125	0.857
145	137	9	9	Yes	135	0.667
155	146	8	8	Yes	145	0.700
165	155	9	9	Yes	155	0.889
175	165	8	8	Yes	165	0.889
185	174	9	9	Yes	175	0.889
195	184	15	15	Yes	185	0.600
205	194	10	10	Yes	195	0.667
215	204	7	7	Yes	205	0.700
225	213	6	6	Yes	215	0.857
235	223	19	19	Yes	225	0.316
245	232	21	21	Yes	235	0.818
255	242	22	22	Yes	245	0.955
265	251	21	21	Yes	255	0.955
275	260	18	18	Yes	265	0.857
285	270	21	21	Yes	275	0.857
295	279	21	21	Yes	285	1.000
305	288	21	21	Yes	295	1.000
315	297	21	21	Yes	305	1.000
325	307	25	25	Yes	315	0.769
335	317	26	25	Yes	325	0.923
345	326	25	25	Yes	335	0.923
355	336	27	25	Yes	345	0.852
365	345	28	25	Yes	355	0.923
375	355	36	25	Yes	365	0.667
385	363	43	25	Yes	375	0.667
395	372	43	25	Yes	385	0.852
405	381	49	25	Yes	395	0.786
415	391	50	25	Yes	405	1.000

N	Unique genes	FDR-significant GO terms	Terms used for Jaccard	Valid enrichment	Previous valid N	Jaccard vs previous valid N
425	401	49	25	Yes	415	1.000
435	411	49	25	Yes	425	1.000
445	421	52	25	Yes	435	0.923
455	431	53	25	Yes	445	0.923
465	440	60	25	Yes	455	0.786
475	450	71	25	Yes	465	0.786
485	460	76	25	Yes	475	0.852
495	468	69	25	Yes	485	1.000
505	476	67	25	Yes	495	1.000

Note. Ranking depths without a significant GO profile were retained for completeness but were not treated as valid profiles for Jaccard comparison. N = 185 is highlighted as the first ranking depth satisfying the reference criterion in the corrected full sweep.

Supplementary Table S3E. Effect of pose-score aggregation on Peptide4 convergence depth

Pose-score aggregation	Convergence-selected N	Top GO terms at selected N
Best-of-four	185	9
Median-of-four	195	21
Mean-of-four	375	25

Note. The primary analysis retained the most favorable of four pose-level PRODIGY estimates. Median- and mean-of-four aggregation preserved strong global receptor-rank correlation but shifted the exact convergence-selected depth, supporting interpretation of N as an operational analysis parameter rather than a biologically invariant cutoff.

Supplementary interpretation

Across the 10 representative peptides, the reference rule identified first qualifying convergence depths between N = 85 and N = 315. The trajectories demonstrate that high local overlap was not uniformly present throughout the ranked lists; instead, the selected depth corresponds to the earliest run of three consecutive valid transitions with J >= 0.80. The corrected Peptide4 full-sweep sensitivity analysis further shows that moderate changes in the Jaccard threshold can preserve the selected depth, whereas more stringent thresholds, longer persistence windows, and alternative pose-score aggregation can shift the first qualifying region. These findings support use of the procedure as a reproducible data-reduction heuristic while arguing against interpretation of the selected N as a unique biological threshold or as validation of individual peptide-protein interactions.
